# Microbiological Profile of Meningoencephalitis Due to Pathogenic Free-Living Amoebae: A Case Series From Kerala, India

**DOI:** 10.7759/cureus.103922

**Published:** 2026-02-19

**Authors:** Pressy Sankar, Anitha P Moorkoth, Sumeeta Khurana, Anuja Mohan, Shameer Vadekkandyil, Shameem A M, Bindu Vadakkayil, Jayesh Kumar P, Shivani Rathor, Sajeeth Kumar K Govindan

**Affiliations:** 1 Microbiology, Government Medical College, Kozhikode, Kozhikode, IND; 2 Medical Parasitology, Postgraduate Institute of Medical Education and Research, Chandigarh, Chandigarh, IND; 3 Internal Medicine, Government Medical College, Kozhikode, Kozhikode, IND; 4 Pediatrics, Government Medical College, Kozhikode, Kozhikode, IND; 5 Preventive Medicine, Government Medical College, Kozhikode, Kozhikode, IND

**Keywords:** acanthamoeba spp, balamuthia mandrillaris, free-living amoeba, india, kerala, meningoencephalitis, naegleria fowleri

## Abstract

Background: Free-living amoebae (FLA) are environmental protozoa that cause rare but often fatal infections of the central nervous system (CNS), collectively manifesting as meningoencephalitis. Data from the Indian subcontinent remain limited. This case series describes the clinical profile, diagnostic challenges, treatment strategies, and outcomes of patients with FLA meningoencephalitis in Kerala, India.

Methods: A retrospective observational study was conducted on 12 patients with microbiologically confirmed FLA meningoencephalitis managed at a tertiary care centre in Kerala between May 2024 and April 2025. Clinical features, laboratory findings, diagnostic modalities, treatment regimens, and outcomes were extracted from medical records and analysed descriptively.

Results: Twelve patients (age range: 6-56 years; median: 32.5 years) were included, with a slight male predominance (58.3%). Over half of the patients resided in Malappuram district. The most common presenting symptoms were fever (91.7%), headache (83.3%), and altered mental status (50%). Cerebrospinal fluid (CSF) analysis showed elevated protein in 75%, reduced glucose in 83.3%, and pleocytosis (median cell count: 157 cells/mm^3^) with either neutrophilic or lymphocytic predominance. Motile trophozoites were detected on fresh CSF wet mount in all cases, while PCR confirmed FLA (*Acanthamoeba *spp.) in 41.7% of patients. All patients received combination antimicrobial therapy including amphotericin B, miltefosine, rifampicin, azithromycin, and fluconazole (with cotrimoxazole substituted for amphotericin B in children). Overall mortality was 41.7%.

Conclusions: FLA meningoencephalitis often presents with non-specific neurological symptoms, making early recognition challenging. Rapid wet mount microscopy of fresh CSF is a pivotal diagnostic tool in resource-limited settings and facilitated early diagnosis in this series. Early initiation of multidrug therapy upon identification of FLA on wet mount microscopy has improved survival. Although mortality remains high, this outcome exceeds the global data reported for *Acanthamoeba* infections and reinforces the value of starting treatment promptly.

## Introduction

Free-living amoebae (FLA), including *Naegleria fowleri, Acanthamoeba *spp., and *Balamuthia mandrillaris*, are widely distributed in soil, fresh or brackish water, dust, and man-made environments, such as swimming pools and air-conditioning systems [1,2]. Although human infections are uncommon, these organisms can cause rapidly progressive and often fatal central nervous system (CNS) infections, ranging from primary amoebic meningoencephalitis (PAM) to granulomatous amoebic encephalitis (GAE) [2].

*N. fowleri* is globally distributed in water and soil. The organism lives in temperatures above 30°C and tolerates temperatures up to 45°C. The life cycle of *N. fowleri* includes three stages: the infective trophozoite, a transient non-dividing flagellate stage, and the cystic stage. The trophozoites measure 10-25 µm and divide by binary fission. The organism contains a single vesicular nucleus with a prominent central nucleolus. *N. fowleri* has been isolated from a variety of fresh and warm water sources worldwide, but not from seawater. Recreational water activities, such as swimming, diving, and surfing, are considered to be common risk factors for infection. Even tap water exposures that can occur as part of religious nasal irrigation are a threat. Asymptomatic children may also be colonised in the nasal mucosa. It is feared that global warming may lead to more widespread occurrences of cases worldwide. Worldwide, PAM caused by *N. fowleri *has been reported more in younger individuals with a history of warm freshwater exposure and has a fulminant, often fatal course [2].

In contrast, *Acanthamoeba* and *Balamuthia* typically cause GAE with a subacute or chronic course, affecting both immunocompromised and apparently immunocompetent hosts, but with poor overall prognosis [2,3]. The *Acanthamoeba *life cycle has two stages: a vegetative trophozoite and a cystic stage. *Acanthamoeba *trophozoites typically measure 15-50 µm. Both the trophozoite and the cyst are uninucleate, with a central nucleolus. The transmission of the amoebae to humans can be through inhalation of cysts or by direct skin contact, followed by haematogenous spread [2]. More recently, *Vermamoeba vermiformis* has also been recognized as a human pathogen [4].

Diagnosis of FLA meningoencephalitis is challenging due to its non-specific clinical presentation, overlap with bacterial and tubercular meningitis, limited clinician awareness, and restricted access to specialized tests, such as polymerase chain reaction (PCR) [5]. Standardised treatment regimens established by randomised trials remain limited. Case fatality rates exceed 90% for *N. fowleri *PAM and are above 70% for *Acanthamoeba *and *Balamuthia *GAE despite aggressive therapy [5]. In India, published reports are fragmented [6,7], and data from southern states are particularly sparse, despite the ecological conditions such as abundant water sources coupled with the tropical climate present in Kerala [8], suggesting a potentially higher risk of disease.

This case series aims to describe the clinico-microbiological characteristics, diagnostic methods, treatment regimens, and outcomes of patients with FLA meningoencephalitis managed at a tertiary care hospital in Kerala, thereby contributing to the growing body of evidence from tropical settings.

## Materials and methods

Study design and setting

An observational, retrospective case series was conducted at Government Medical College, Kozhikode, Kerala, India. All patients diagnosed with FLA meningoencephalitis between May 2024 and April 2025 and meeting the inclusion criteria were enrolled. A waiver of informed consent was obtained from Instituotional Ethics Committee (GMCKKD/RP2025/IEC/307). Patient anonymity was strictly maintained throughout the study. Patient details were collected from the laboratory and medical records.

Patient selection

Inclusion criteria comprised (i) clinical features consistent with meningoencephalitis (e.g., fever, headache, neck stiffness, altered sensorium, and seizures) and (ii) microbiological confirmation of FLA infection through cerebrospinal fluid (CSF) wet mount microscopy and/or molecular detection. Patients with alternative confirmed aetiologies were excluded.

Diagnostic procedures

CSF samples were collected under sterile conditions and processed immediately. For optimal detection of viable amoebae, wet mount microscopy was performed within 30 minutes of sample receipt to assess trophozoite motility and morphology. Routine CSF tests included total and differential leukocyte counts, protein and glucose levels, Gram stain, and bacterial culture to rule out common bacterial pathogens.

The CSF samples after initial investigations were stored at 4-8°C in the refrigerator. All CSF samples were transported to the Postgraduate Institute of Medical Education and Research (PGIMER), Chandigarh, for molecular testing. The cold chain was maintained during transit with ice packs, and the samples were secured with triple-layer packaging in accordance with biosafety guidelines. PCR assays targeting amoebic DNA were performed off-site at the institute for species-level identification.Molecular assays included conventional pan-genus FLA and genus-specific PCR for *Acanthamoeba, Naegleria*, and *Balamuthia* species and real-time PCR assays. The *Acanthamoeba*-specific primer pair used was the forward primer (5’-GGCCCAGATCGTTTACCGTGAA-3’) and the reverse primer (5’-TCTCACAAGCTGCTAGGGAGTCA-3’). The conventional PCR amplified products were sequenced for confirmation [9,10]. There was an average delay of 10-14 days in molecular diagnosis, as no in-house testing facility was available at that time. 

Treatment protocol

All adult patients were treated with a standardized multidrug regimen as per state guidelines, comprising conventional amphotericin B, miltefosine, rifampicin, azithromycin, and fluconazole for a total duration of 28 days, unless limited by intolerance or death [11,12]. In paediatric patients, cotrimoxazole, miltefosine, rifampicin, azithromycin, and fluconazole were given according to institutional protocol [13]. Supportive care included ICU management as indicated, seizure prophylaxis, and management of raised intracranial pressure with anti-edema measures, such as hypertonic saline and intravenous mannitol. The criteria for ICU admission included the presence of hemodynamic instability, recurrent or refractory seizures, and a depressed level of consciousness as reflected by a low Glasgow Coma Scale score.

Data collection and analysis

Demographic variables, epidemiological factors (including water exposure), comorbidities, clinical manifestations, laboratory findings, therapeutic details, and outcomes were retrieved from medical records. Data were summarized using descriptive statistics, with categorical variables expressed as frequencies and percentages, and continuous variables as medians with ranges.

## Results

Demographic and epidemiological characteristics

Twelve patients, including two children, fulfilled the inclusion criteria during the study period. The median age was 32.5 years (range: 6-56 years), with seven males (58.3%) and five females (41.7%). Seven patients (58.3%) were from Malappuram district, and the remaining five (41.7%) were from Kozhikode district.

Six patients (50%) reported a plausible history of water exposure, including well cleaning, river bathing, or ritual nasal rinsing. The other six patients did not reveal any obvious history of water exposure. Three patients (25%) had significant risk factors, two with diabetes mellitus and one who was pregnant.

Clinical manifestations

Fever was the most frequent symptom, affecting 11 patients (91.7%), followed by headache in 10 (83.3%) and altered sensorium in six (50%). Additional symptoms included vomiting in four (33.3%), photophobia in two (16.7%), seizures in two (16.7%), and altered behaviour in two (16.7%). One patient presented with focal neurological deficit (right-sided hemiparesis) and another patient with dysarthria.

Ten patients (83.3%) presented with an acute onset (one to nine days) and rapid neurological deterioration, which was clinically consistent with PAM, and two patients (16.6%) had an insidious, subacute course, suggestive of GAE (Table 1).

**Table 1:** Demographic and clinical characteristics of patients with FLA meningoencephalitis (n=12) *Risk factors included diabetes mellitus (n=2) and pregnancy (n=1). FLA: Free-living amoebae

Characteristic	Number (%) or Median (Range)
Age, years	32.5 (6–56)
Female	5 (41.7%)
Male	7 (58.3%)
Malappuram district	7 (58.3%)
Kozhikode district	5 (41.7%)
History of any water exposure	6 (50%)
Risk factors*	3 (25%)
Fever	11 (91.7%)
Headache	10 (83.3%)
Altered sensorium	6 (50%)
Vomiting	4 (33.3%)
Altered behaviour	2 (16.7%)
Photophobia	2 (16.7%)
Seizures	2 (16.7%)
Focal neurological deficit	1 (8.3%)
Dysarthria	1 (8.3%)

CSF findings

CSF analysis showed abnormalities compatible with meningoencephalitis in all cases. Median CSF protein was 125 mg/dL (range: 19-1,268 mg/dL), with elevated protein (>45 mg/dL) in nine of 12 patients (75%). CSF glucose was reduced in 10 patients (83.3%), and all specimens showed pleocytosis (median cell count: 157 cells/mm^3^), with equal distribution between neutrophilic (50%) and lymphocytic (50%) predominance.

**Figure 1:**
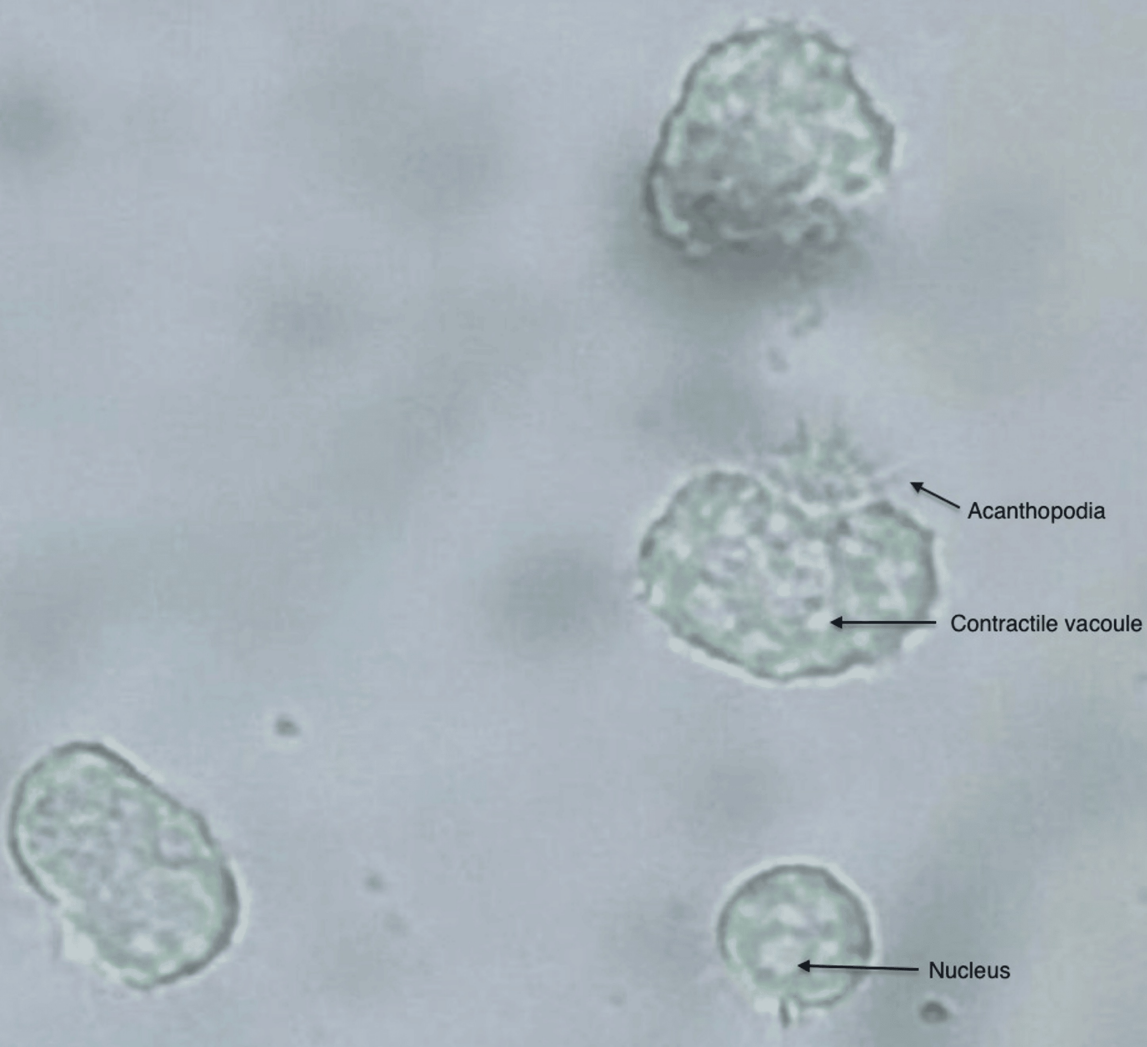
Acanthamoeba trophozoite *Acanthamoeba *trophozoite in wet mount showing characteristic fine thorn-like acanthopodia, contractile vacuoles, and nucleus under 40X magnification with a light microscope.

Wet mount microscopy of freshly collected CSF demonstrated motile trophozoites in all 12 patients (100%). The trophozoites typically displayed sluggish amoeboid movement, acanthopodia, granular cytoplasm, and a distinct central nucleus with a prominent nucleolus and a contractile vacuole, consistent with *Acanthamoeba* morphology (Figure 1) [2]. In a subset of cases, abrupt jet-like propulsive movements were seen [14].

Molecular diagnosis and correlation

PCR-based assays confirmed *Acanthamoeba *spp. in five of 12 patients (41.7%). Seven samples remained PCR-negative despite clear microscopic evidence of FLA, and neither *Naegleria *nor *Balamuthia* DNA was detected in any sample.

Treatment and outcomes

All 12 patients received the standardized five-drug regimen (amphotericin B, miltefosine, rifampicin, azithromycin, and fluconazole), with cotrimoxazole used instead of amphotericin B in two paediatric cases, upon identification of free living amoebae by morphology on wet mount microscopy. The treatment was continued for a minimum of 28 days to ensure that all predefined criteria for cure were met. The criteria included resolution of presenting symptoms and neurological signs, normalisation of CSF cell counts and biochemical parameters, demonstration of a negative wet mount microscopy for amoebae, and resolution of radiological findings, if any. Clinical improvement was documented in seven patients (58.3%), all of whom were diagnosed early by microscopy and received prompt therapy.

Five patients (41.7%) died. Two patients with diabetes mellitus and one pregnant woman were among the non-survivors, suggesting an adverse prognostic impact of underlying conditions. One patient who initially improved subsequently experienced a relapse after five months. Despite the prolonged therapy, she succumbed to the illness. In the other four patients, the duration from onset to death was 7-10 days. All the survivors had full neurological recovery (Table 2).

**Table 2:** CSF findings, treatment, and outcomes

Parameter	Result
Elevated CSF protein (>45 mg/dL)	9/12 (75%)
Low CSF glucose	10/12 (83.3%)
Neutrophilic pleocytosis	6/12 (50%)
Lymphocytic pleocytosis	6/12 (50%)
Wet mount positive for trophozoites	12/12 (100%)
PCR positive for FLA (*Acanthamoeba *spp.)	5/12 (41.7%)
Standardized multidrug regimen given	12/12 (100%)
Survival	7/12 (58.3%)
Mortality	5/12 (41.7%)

## Discussion

This series describes 12 patients with FLA meningoencephalitis from Kerala. These infections, while uncommon, are critical due to their often-fatal outcomes [15]. In this cohort, the observed age range was 6-56 years (median: 32.5 years) with minor male predominance, while the established global epidemiological trends typically report a higher incidence among younger males, particularly in cases of PAM [3].

This difference may reflect environmental and social factors unique to our geographic region and the presence of both PAM and GAE phenotypes within the study population.

*Acanthamoeba* species, the predominant pathogen in this series, are environmental organisms thriving widely in natural bodies of water and soil, as well as in manmade water systems and hospital environments. Their wide distribution explains why only half of our patients reported explicit water contact, suggesting multiple exposure routes [16].

Despite FLA infections being historically linked to immunosuppression, most patients in this series were apparently immunocompetent, underscoring the fact that FLA can cause severe disease in both immunocompetent and immunocompromised individuals [5]. The observation that all patients with significant comorbidities or pregnancy experienced fatal outcomes further suggests that underlying medical conditions may exacerbate disease severity and negatively impact prognosis in FLA meningoencephalitis [15].

Clinically, these amoebic infections produced symptoms largely indistinguishable from other meningoencephalitides, complicating early diagnosis. CSF examination typically showed elevated protein and decreased glucose along with pleocytosis, but with no single diagnostic biochemical signature. This variability underscores the challenges in differentiating FLA infections from other causes of meningoencephalitis based on routine CSF parameters alone [17].

The detection of motile trophozoites on wet mount microscopy proved valuable as a rapid diagnostic tool in our setting, with 100% positivity in our series. This supports its value for routine wet mount examination of CSF within 30 minutes of sample collection in cases of suspected meningoencephalitis, particularly in endemic regions [17].

Under wet mount microscopy, *N. fowleri *trophozoites exhibit amoeboid morphology and progress in a distinctly sinuous manner. Each trophozoite contains a prominent nucleus with a large central nucleolus, as well as a contractile vacuole that forms and empties cyclically. The cyst formation is not seen in human tissue [18,19].

The trophozoites of *Acanthamoeba *species exhibit slender, spine-like extensions known as acanthopodia, which are continually extended and retracted. Under unfavourable conditions, these amoebae encyst, producing a double-walled structure; the outer ectocyst typically displays a rippled or wrinkled appearance, whereas the inner endocyst may be polygonal, oval, or round. Within the trophozoite, a prominent contractile vacuole cyclically appears and disappears, with a cycle of approximately 45-50 seconds. In some instances, the trophozoites demonstrate abrupt, forceful movements [14]. The cysts may be frequently seen in CSF or brain tissue in patients with *Acanthamoeba *meningoencephalitis [1,20,21].

In this series, fresh CSF wet mount microscopy within 30 minutes of sample collection demonstrated motile trophozoites in all patients and was the key to rapid diagnosis. The 100% positivity of wet mounts highlights their value in resource-limited settings, especially when molecular tests are unavailable or delayed.

PCR analysis detected subgenic 18S ribosomal DNA (18S rDNA) sequences that are highly specific to the *Acanthamoeba *genus in all PCR-positive samples. 18S rDNA gene sequence analysis has been proposed as a recent method for molecular identification of *Acanthamoeba* [9,10]. However, PCR assays confirmed *Acanthamoeba* infection in only 41.7% of cases, despite strong microscopic evidence of FLA. Possible explanations for a negative PCR result include DNA degradation during transport owing to prolonged transit time and consequent delayed processing at the referral laboratory, low organism load, and primer mismatch for non-target FLA species [2,22-24]. It also highlights the ongoing need for improved molecular assays with broad primer coverage and robust sample handling workflows, along with local laboratory capacity building.

The multidrug regimen used in this cohort, comprising amphotericin B, miltefosine, rifampicin, azithromycin, and fluconazole (with cotrimoxazole in children), was associated with a survival rate of 58.3%, which appears more favourable than historical reports [11,12,25]. Early diagnosis by microscopy and prompt initiation of combination therapy likely contributed to improved outcomes [5,15]. Standard supportive measures, including intracranial pressure management, were provided uniformly as part of routine care and likely aided clinical stabilization. Additionally, the case series encompassed probable infections by distinct FLA, which exhibit variable clinical trajectories and survival outcomes.

The relapse case presented with headache and seizure episodes one month after discharge. She was treated for a total duration of 68 days and was on follow-up. However, after three months, she developed another episode of seizure and succumbed immediately on admission. The occurrence of relapse in this case emphasizes the need for careful post treatment surveillance, follow-up neuroimaging, and rigorous, prolonged therapy, if indicated [1,26]. The criteria for patient discharge as per institutional protocol included complete clinical recovery with microscopic clearance of the organism and resolution of radiological findings.

Future directions should include factors such as strengthening in-house molecular diagnostic capabilities, evaluating adjunctive interventions such as intrathecal amphotericin and therapeutic hypothermia, and conducting environmental surveillance to better define exposure risks in endemic areas. Multicentric collaborative studies from India and other tropical regions are needed to refine diagnostic algorithms and therapeutic protocols [5,15,25].

Limitations

This study is limited by its relatively small sample size and single‑centre design. Wet mount microscopy, being a qualitative and observer‑dependent technique, introduces variability related to examiner expertise, and its importance, as highlighted in this case series, may not be generalizable to resource‑rich settings equipped with in-house molecular diagnostic facilities. Molecular confirmation was not available for all cases, which may affect diagnostic accuracy. Additionally, the absence of long‑term follow‑up limits assessment of recurrence and sustained treatment outcomes.

## Conclusions

Free-living amoebic meningoencephalitis presents with non-specific neurological symptoms, which makes the diagnosis challenging. A high degree of clinical suspicion, coupled with early wet mount microscopic examination of freshly collected CSF samples, allows reliable identification of amoebae in resource-limited settings. Although the mortality remains high, early initiation of combination antimicrobial therapy has improved patient outcomes. This series highlights the importance of considering FLA in the differential diagnosis of meningoencephalitis in tropical regions.
